# Evaluation of INSTAND e.V.’s external quality assessment for C-reactive protein and procalcitonin

**DOI:** 10.1371/journal.pone.0221426

**Published:** 2019-08-16

**Authors:** Nathalie Wojtalewicz, Ingo Schellenberg, Klaus-Peter Hunfeld

**Affiliations:** 1 INSTAND e.V., Society for Promoting Quality Assurance in Medical Laboratories, Duesseldorf, Germany; 2 Institute of Bioanalytical Sciences (IBAS), Center of Life Sciences, Anhalt University of Applied Sciences, Bernburg, Germany; 3 Institute for Laboratory Medicine, Microbiology & Infection Control, Northwest Medical Centre, Frankfurt/Main, Germany; Inselspital Universitatsspital Bern, SWITZERLAND

## Abstract

**Background:**

The purpose of this paper was to analyze the general diagnostic strength and performance of *in vitro* diagnostics for C-reactive protein and procalcitonin based on the results of external quality assessment schemes (EQAs).

**Methods:**

We analyzed qualitative and quantitative data on both markers collected by the Society for Promotion Quality Assurance in Medical Laboratories (INSTAND e.V.) from 20 EQAs. The C-reactive protein evaluation was method-specific and the procalcitonin evaluation manufacturer-specific (pseudonymized). Coefficients of variation were determined in order to evaluate interlaboratory comparability and the performance of individual laboratories during the analyzed period was examined.

**Results:**

Overall most of our participants were able to correctly distinguish the positive from the negative samples, but we occasionally observed also false-positive results for the immunological detection of C-reactive protein. For the semi-quantitative results of C-reactive protein we observed an overall median difference below 5% except for dry chemistry methods (≤ 21%). For procalcitonin two manufacturer collectives showed a good comparability, while one manufacturer detected up to 42% higher results. The coefficients of variation are promising for both analytes even though they surpass the manufacturer’s indication for some collectives. The performance of individual laboratories during the analyzed period was more stable for C-reactive protein than for procalcitonin.

**Conclusion:**

*In-vitro* diagnostic testing for C-reactive protein and procalcitonin showed promising results in our EQAs but still further improvements are needed. We recommend stepping up research on reference measurement methods for both parameters to possibly enhancing the accuracy and diagnostic strength of such assays.

## Introduction

Laboratory testing is an important tool in modern medical diagnostics. The identification and detection of clinically useful marker molecules has a particularly strong impact on patient management [1]. Using circulating biomarkers, such as C-reactive protein (CRP) and procalcitonin (PCT), to accurately diagnose, manage and treat infection-induced inflammatory diseases, like sepsis [2] and pneumonia [3], is a highly researched medical field.

CRP is an acute-phase protein that is produced in the liver in response to tissue injury and inflammatory events of all types of etiology [4–6]. It has been widely used in European emergency departments (ED) as a biomarker to screen for the presence of inflammation or infectious disease [7], particularly in pediatrics [8].

PCT, a precursor of calcitonin, is a hormone that is released in response to significant systemic inflammation, mainly following bacterial, fungal and parasitic infection [9].

Serial measurements of both proteins are commonly used to monitor the effectiveness of antimicrobial therapy and in follow-up measurements of infection [10, 11]. Therefore, it is crucial that the protein’s test methods work as accurately as possible in order to ensure that such clinical biomarkers retain their usefulness in patient management.

The use of internal and external quality control programs is an important tool to continuously monitor the quality of such laboratory measurements, not only to ensure the general quality of laboratory diagnostics and, thus, the health of the patient, but also due to an increasing pressure to reduce costs by improving quality [12]. The Society for Promoting Quality Assurance in Medical Laboratories (INSTAND e. V.) has managed external quality assessment schemes (EQAs) in the field of laboratory diagnostics since 1968 and is one of only three officially appointed reference institutions in Germany.

Despite the high number of multi-center trials and pro- and retrospective analyses of the diagnostic value of CRP and PCT, studies comparing the diagnostic quality of both parameters over a longer period of time are still pending. In this paper, we analyze the quantitative and qualitative results obtained from EQAs conducted over a five-year period for CRP and ten-year period for PCT. In addition to reporting on passing rates and measurement quality, coefficients of variations are used for interlaboratory comparability of the test results.

## Methods

### External quality assessment procedures at INSTAND e.V

The data from the EQAs was obtained from regular EQAs in Europe. Each EQA participant receives one positive and one negative sample per analyte. Participants can report the qualitative and quantitative results of the CRP detection, while, in the case of PCT, semi-quantitative results can also be provided as well as a diagnostic comment on each sample (data on diagnostic comments not analyzed).

To analyze CRP testing, the EQA expert formed method-specific collectives and took into consideration an evaluation area of 20% around the median for the quantitative results in accordance with the guidelines for medical laboratories issued by the German Medical Association [13]. The evaluation for quantitative PCT was manufacturer-specific with an evaluation range that changed from 30% to 25% within the timeframe. With respect to the qualitative results, the participants had to indicate whether the samples were positive, borderline or negative; for the semi-quantitative results they could report ranges.

### Sample material

The positive samples consist of pools of retained native patient materials, while the negative samples are taken from voluntary blood donors. The samples were tested negative for HIV, HBV and HCV. No stabilizing additives were added [14]. Homogeneity of each sample batch was tested according to DIN EN ISO/IEC 17043:2010–05 before the samples were used in the corresponding EQA [15]. The patient’s informed written consent is available for the project. A positive vote from the ethic committee of the Goethe University of Frankfurt (Main) has been obtained for samples of voluntary blood donors, whereas in Germany no ethics vote is necessary for the use of retained patient samples.

### Data evaluation and statistics

We evaluated 20 PCT EQAs that were organized by INSTAND e.V. between 2008 and 2017 and 20 CRP EQAs conducted between 2013 and 2017. The CRP evaluation was method-specific (S1 Table), whereas the PCT evaluation was manufacturer-specific (S2 Table). The manufacturers were pseudonymized and the codes are listed at https://www.instand-ev.de/no_cache/en/eqas-online/service-for-eqa-tests/. The groups can be filtered for EQA 320 (PCT) or EQA 322 (CRP). A term and analyte must then be selected. The manufacturer codes can be found below the statistical data in the box “reagent” (“r”).

Values that exceeded the calibration curve by more than 20% were excluded from the analysis because they were most likely transcription errors or methodical outliers. When analyzing the method and manufacturer collectives, we corrected obvious errors resulting from sample swaps so they would not distort the general quality of the test results. In order to evaluate the qualitative performance of the EQA participants, no corrections were made when analyzing whether the positive sample was correctly identified. We used cut-off values of 5 mg/l for CRP [16, 17] and 0.5 ng/ml for PCT [18, 19].

Finally, we compared the results of individual laboratories with the median of all participants to evaluate performance over several EQAs (S3 Table). We chose the participants based on their collectives and made sure that they used the same reagents for all EQAs wherever possible.

Basic statistical analyses were performed using SigmaPlot13 from Systat Software Inc. (Erkrath, Germany) and jmp from SAS Institute (Cary, North Carolina, USA).

### Generation of images

The overlay images were generated using the Gnu image manipulator software 2.8.1.

## Results

### Initial diagnosis and passing rate

**Fig 1:**
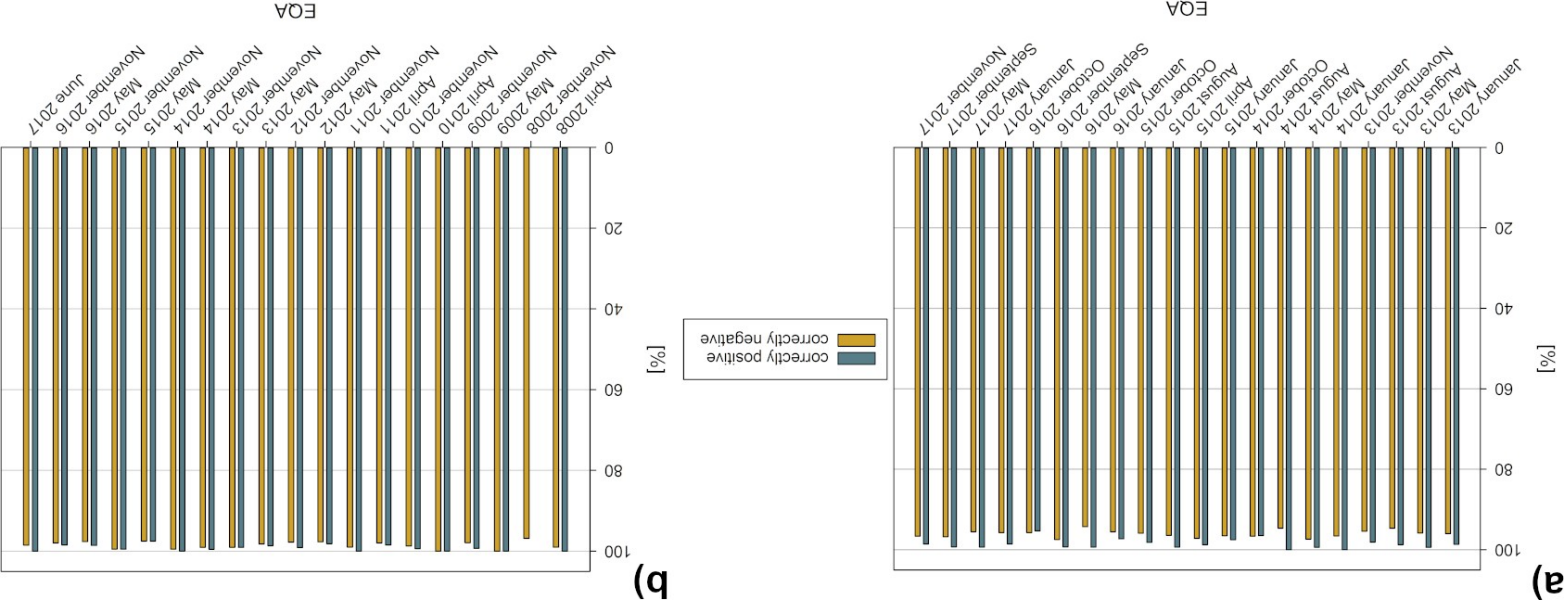
Correct discrimination between positive and negative samples for CRP (a) and PCT (b).

We examined 20 EQAs for both analytes. Each CRP EQA involved around 370 laboratories, while the number of participants of the PCT EQAs increased from 100 to around 200. In terms of qualitative results, over 95% of the participating laboratories were able to correctly discriminate between positive and negative samples when cut-off values of 5 mg/l for CRP [16, 17] and 0.5 ng/ml for PCT [18, 19] were applied. The primary source of error was sample swapping. In November 2008 there was no negative sample present for PCT (Fig 1).

**Fig 2:**
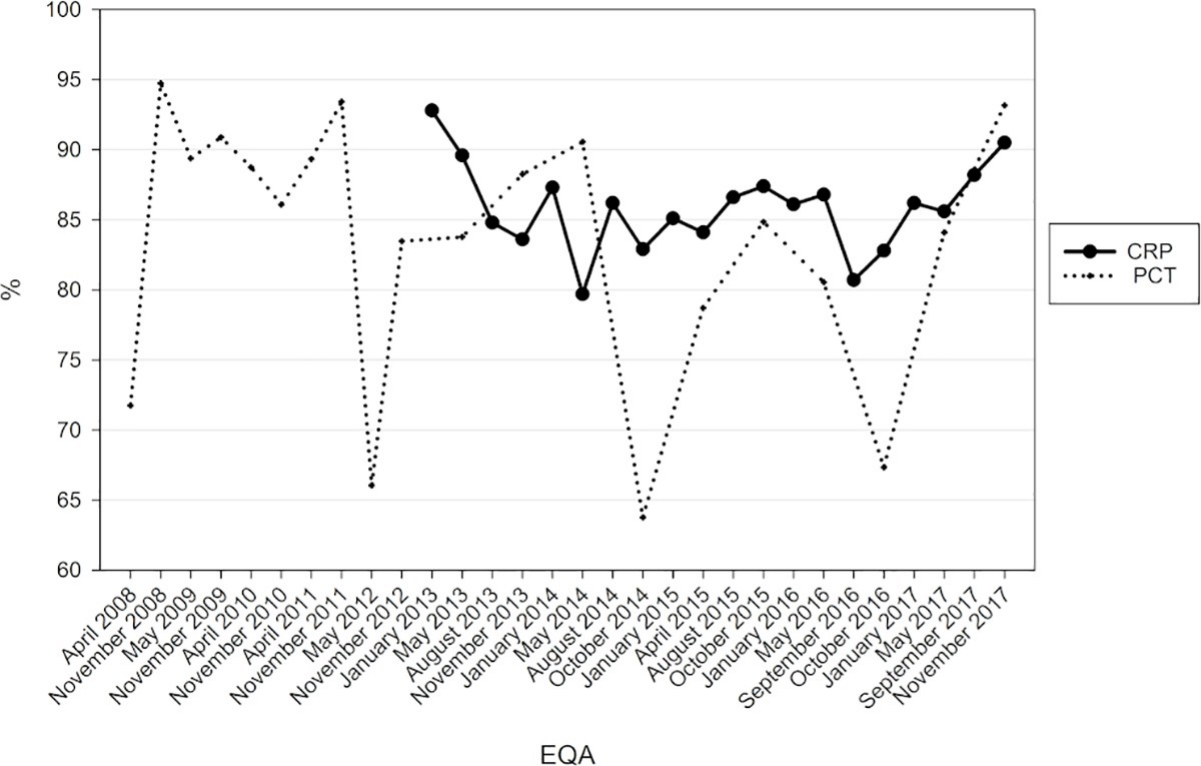
Passing rates of the EQAs for c-reactive protein (CRP) and procalcitonin (PCT).

The general passing rates for the quantitative results were, however, lower, ranging from 80% to 90% for CRP and from 64% to 90% for PCT (Fig 2).

### Analysis of method-based distribution for quantitative CRP results

In the next step, we evaluated the method-specific distribution of the quantitative CRP results. Since more than 90% of our participants used a method based either on immunoturbidimetry, nephelometry, immunological detection or dry chemistry, we focused on these four collectives when evaluating the timelines and analyzing the coefficient of variation.

**Fig 3:**
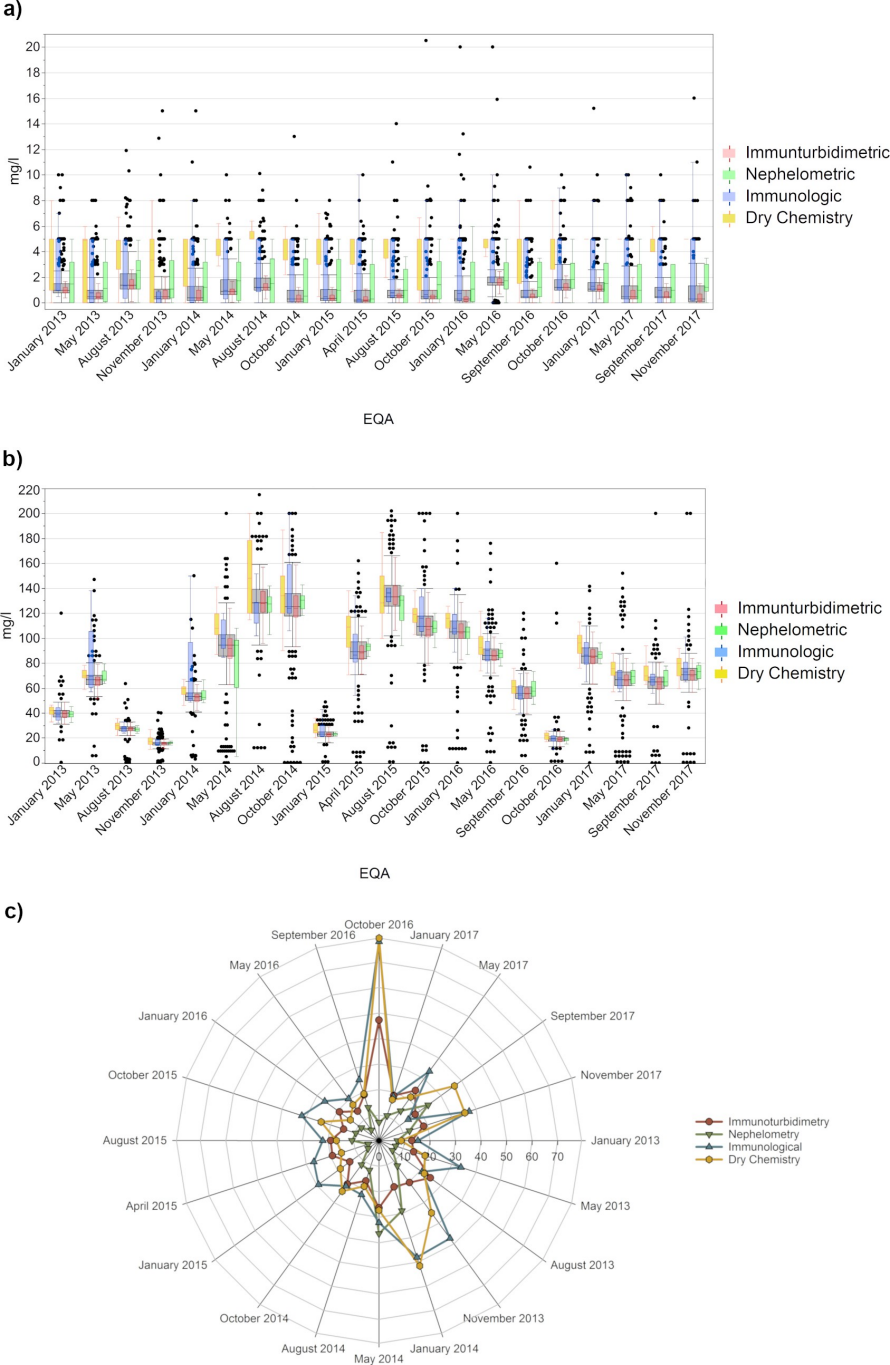
Analysis of the EQA results for CRP. (a) for the negative sample, (b)for the positive sample and (c) the interlaboratory comparability based on the coefficient of variation for the four main methodic test principles. The grey boxes display all results from the respective RRT, and the results obtained with the specific method-based collectives are illustrated as colored box plots with whiskers reaching from 1st quartile—1.5*(interquartile range) to 3rd quartile + 1.5*(interquartile range). An immunoturbidimetric approach is colored red, nephelometric methods green, immunological tests blue and a dry chemistry approach orange.

In the negative samples (Fig 3A), nearly all collectives remained below the defined reference are for a non-septic patient (5 mg/l) [16, 17]. The immunoturbidimetric collective exhibited the lowest results (mostly below 2 mg/l) and the lowest scatter of values. Up to 25% of the participants of the immunological and the dry chemistry collectives overestimated their results from time to time.

Starting at the end of 2016, the four methods began aligning better in terms of the results for the positive samples (Fig 3B). The median values of three of the four collectives differed by under 5%. The only exception was the dry chemical approach, which led to 7 to 20% higher median test results. Value distribution for the immunological and dry chemical methods tended to be very wide in the past, especially in 2014, however this discrepancy has decreased since 2016. As a result, we observed a clear alignment of all test methods used in the EQAs.

To get a better impression of the interlaboratory comparability of the different methods, we calculated coefficients of variation (CV) for each collective (Fig 3C). The nephelometry collective achieved the best results, with CVs primarily below 20% with only three exceptions. Interestingly, this is the only collective with low CV results in October 2016, while the other methods clearly exceeded a CV of 40%. The participants using immunoturbidimetry usually exhibited slightly higher CVs than those using nephelometry but remained around or below 20% most of the time.

For the other two collectives, a wider spread of box plots already indicated a poorer interlaboratory comparability of these methods, with CVs confirming this suspicion. This especially remains true for the participants using immunological methods whose CVs were just under 20%.

### Analysis of manufacturer-based distribution for PCT

We analyzed the PCT data based on manufacturer collectives. Seventy-five percent of participants used test systems from one of the three manufacturers r014, r082 and r149. The other 25% used various tests from a variety of manufacturers and are therefore consolidated under “other manufacturers”.

**Fig 4:**
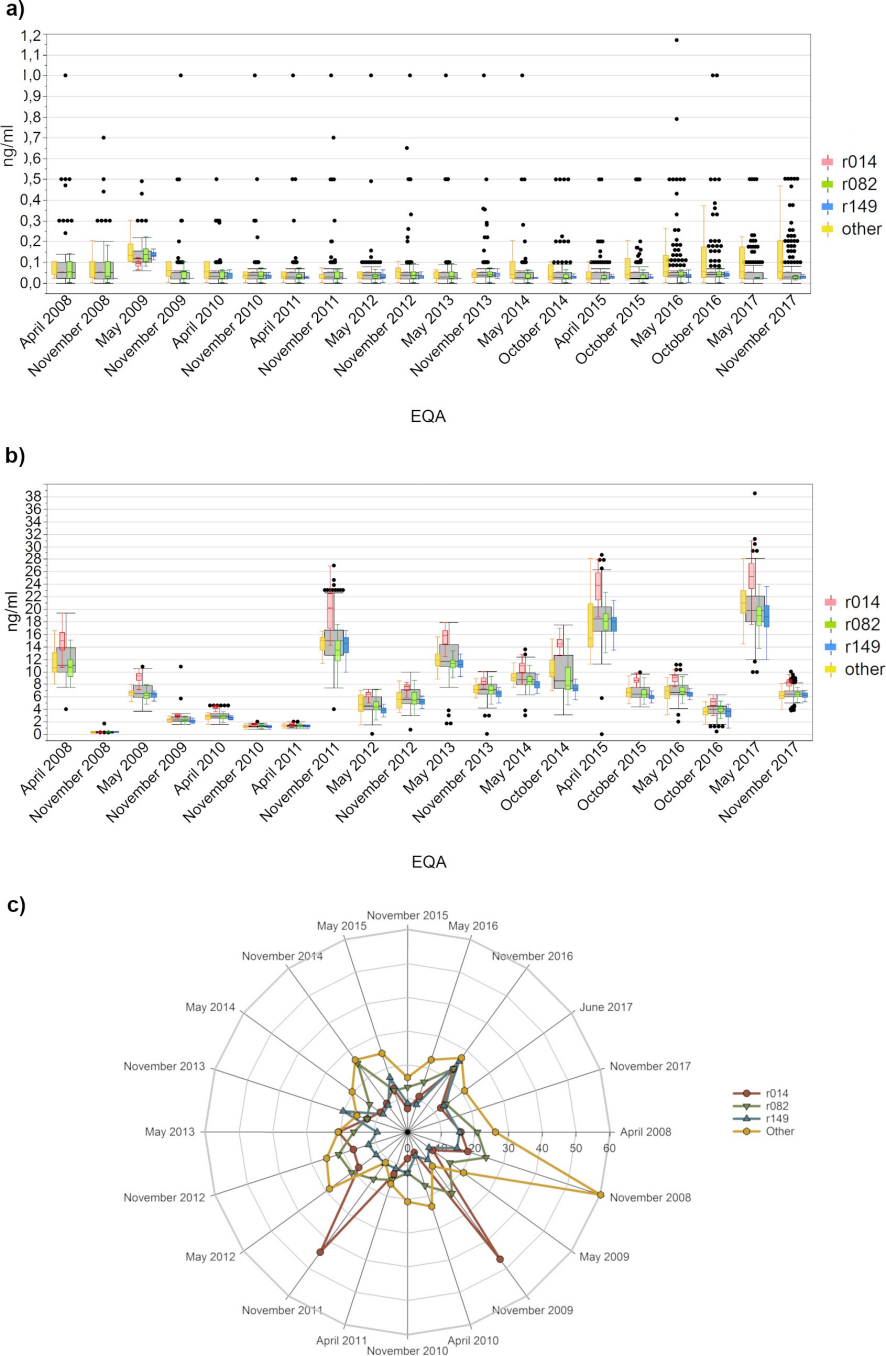
Analysis of the EQA results for PCT. (a) for the negative sample, (b)for the positive sample and (c) the interlaboratory comparability based on the coefficient of variation based on the coefficient of variation (c) for the four main methodic test principles. The grey boxes display all results from the respective RRT, and the results obtained with the specific manufacturer collectives are illustrated as colored box plots with whiskers reaching from 1st quartile—1.5*(interquartile range) to 3rd quartile + 1.5*(interquartile range). r014 is colored red, r082 green, r149 blue and other manufacturers orange.

For the negative samples (Fig 4A), more than 90% of all participants showed values below the clinical cut-off for the diagnosis of a systemic infection (0.5 ng/ml) [18, 19]. The collective “other manufacturers” displayed the widest value distribution.

The situation was completely different for the positive samples (Fig 4B). The manufacturer collective r014 clearly exceeded the other three groups. The median of this collective differed by 17–33% from the median values of the other participants with one exception in October 2014, where they were even 42% apart. The collectives r082 and r149 were well aligned and presented nearly identical value distributions, although the r149 collective seemed to show a slightly better interlaboratory comparability (Fig 4C). The r149 collective stayed below a CV of 20% for all but one EQA. The collectives r014 and r082 were relatively comparable and their CVs often remained below 20%. Until October 2014, their CVs were usually up to 10% worse than those of r149, but since April 2015 the interlaboratory comparability of the three main collectives has nearly aligned. The higher CVs of the “other manufacturer” collective were due to the large number of different *in vitro* diagnostics providers within this group. Nevertheless, all collectives show the same trends for the CVs for each sample.

### The performance of individual participants in several EQAs

**Fig 5:**
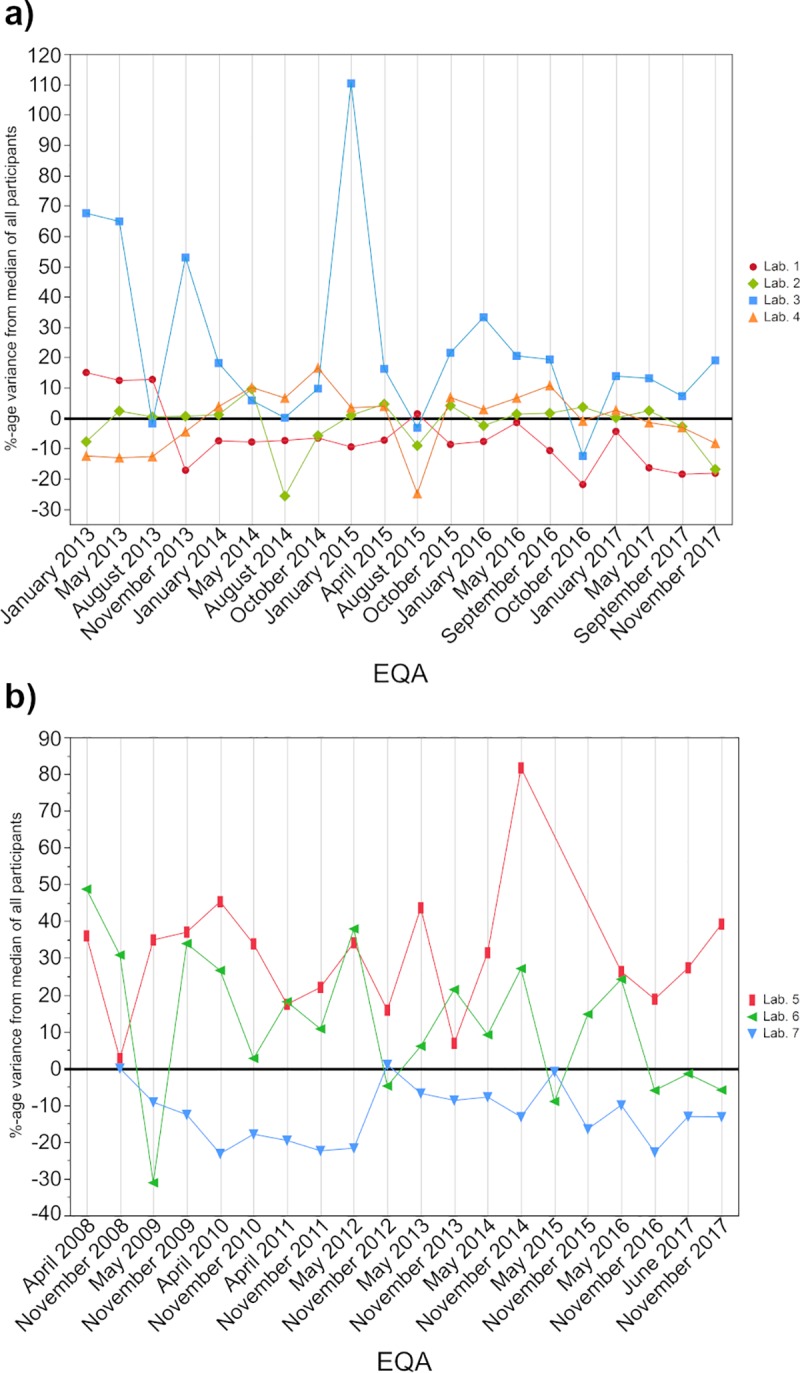
**Comparison of the percentage deviation of single laboratories to the corresponding EQA median results for CRP (a) and PCT (b).** For CRP, laboratory 1 used an immunoturbidimetric approach, lab. 2 used nephelometry, lab 3 immunologic methods and lab. 4 a dry-chemistry approach. In case of PCT, lab 5 used a test system from r014, lab 5 from r082 and lab 7 from r149.

As a next step, we wanted to see whether the observed differences between the collectives remained constant for several independent measurements conducted in individual laboratories and whether external quality control had any influence on the general performance of these laboratories. We compared the CRP values of four different participants (Fig 5A) and the PCT values of three participants (Fig 5B) to the general median values of all participants combined. The participants were chosen based on their collectives and their continuous usage of the same method or reagent for all EQAs wherever possible.

In the case of CRP, three of the four laboratories showed value fluctuations that were to be expected based on the CVs observed in Fig 2C. The results also revealed a wavelike distribution around the median over the observed time period. Laboratory 3 (immunological methods) deviated greatly from the overall median at the beginning of 2013, but its values were more consistent in later EQAs, with one exception in January 2015.

For PCT, the manufacturer-dependent differences observed in Fig 3B are clearly visible for the three observed laboratories. Participant 5 (r014) showed higher values than the other two laboratories. Interestingly, the results for laboratories 6 (r082) and 7 (r149) only align in some EQAs, although their manufacturer collectives showed a good comparability (Fig 4B). Furthermore, the wavelike patterns of the results usually remain above or below the average median of all participants and are not distributed around it, as we observed for CRP.

## Discussion

The specific diagnosis, clinical differentiation and reliable monitoring of inflammatory and infectious diseases continues to reveal a plethora of challenges in daily practice. Therefore, researchers have made many efforts to identify one or more biomarkers that could help to better distinguish inflammation and infection and to effectively monitor the effectiveness of therapy during antibiotic treatment [20, 21]. The two most prominent proteins, CRP and PCT, are used as routine markers in clinical practice, but their use as inflammation and infection markers and as possible markers for the clinical success of the antibiotic treatment is still controversial [21–23].

This paper is the first to analyze the general performance of *in vitro* diagnostics for CRP and PCT in external quality assessment schemes (EQAS). The analysis offers an independent evaluation of the overall performance of the corresponding test methods since all participants in an EQA receive the same sample material. One of the EQA’s tasks is to distinguish between positive and negative samples. We used cut-off values of 5 mg/l for CRP, since the reference range for a non-septic adult is < 5 mg/L [16, 17] and 0.5 ng/ml for PCT since this concentration is used in various algorithms for monitoring sepsis [18, 19].

Fortunately, over 95% of our participants were able to correctly diagnose the positive and negative samples. However, the quantitative results are of critical importance as both markers are used for follow-up in the diagnosis and stewardship of various infectious diseases. Unfortunately, the quantitative results are not as good as the qualitative ones.

For CRP, the value distribution of all participants is comparable, with overall median differences below 5%. The dry chemical method is the only exception as it yielded median values that were between 7 and 21% higher. In addition, the immunological and dry chemical approaches showed false positive results up to 25% of the time for the negative sample. These findings correlate with published results of immunoassays with relatively high false-positive rates due to non-specific binding [24].

Although we observed relatively small box plot ranges, the CVs in our analysis still differ from the manufacturer’s specifications. Nearly all suppliers indicate an interlaboratory CV of below 10% or even 5% for their test systems. In our EQAs, only the nephelometric collective was able to achieve these low deviations, whereas the immunoturbidimetric and the dry chemical methods usually had a CV of around 20% and the immunological methods a CV of 20 to 30%.

Even if these CV are lower than the ones, we observed previously for allergy diagnostics [25, 26], they could have a diagnostic impact: A decrease in CRP of at least 25% compared to the patient’s previous test result is considered a good indicator of sepsis resolution. With a variation potential of up to 30% for the immunological methods, the clinically relevant decline might be indicated by the uncertainty the tests alone. This shows that there is still room for improvement with respect to interlaboratory comparability, especially for the immunological methods, although the general development of the results is promising. Nevertheless, we would currently recommend excluding immunological methods from progress monitoring of serious infections, since the standard tests are more sensitive and reliable.

A vast number of publications compare different PCT test systems, but the results are controversial since a good comparability of two test systems is either confirmed [27] or refuted [28, 29]. These differences are often argued as being based on the patient pool selected for each analysis.

In our publication we were able to show that two manufacturer collectives align quite nicely, while the collective r014 has median values that are, on average, 27% higher for each EQA (Fig 3B). Since a decline in PCT levels by at least 30% is a good indicator for the improvement of the inflammation [30], our results reinforce the statement by Schuetz et al. that PCT levels “should always be discussed in the context of the clinical setting and the technical performance of the corresponding test system”[30, 31]. This statement gains further weight as we have observed a change in the test system of several participants in recent years. If laboratories keep these old devices as standby units, this might result in serious outcomes for the patient unless the device-specific measurement ranges are considered and communicated to the attending physician.

Fortunately, this difference did not affect the detection of the negative samples, where many participants identified values below 0.1 ng/ml. Several algorithm-guided PCT studies define this concentration as the point where an infection is very unlikely [31]. Our data shows that the sensitivities of the individual test systems are sufficient to reliably detect this threshold.

The manufacturers of the PCT assays specify different CV values for the interlaboratory comparability of their test systems, with 16% being the highest. We observed that all three primary collectives usually remained below this threshold, however, from time to time the CVs of each collective peaked as high as 25%. These peaks do not correlate with the PCT concentrations and we could not see any differences connected to the batch of the test kits our participants used. A matrix effect is unlikely, since our samples are native serum samples without any additives. Furthermore, our samples are thoroughly tested for homogeneity according to DIN EN ISO/IEC 17043:2010–05 [15] and we could not observe any abnormality in the corresponding samples.

Taken together, we observed a better reproducibility of the CRP measurements compared to the PCT values. This may be due to the fact that the clinically relevant analyte concentrations differ by several orders of magnitude (CRP [mg/L]; PCT [ng/ml]). Furthermore, CRP has been known for a longer period of time and *in vitro* diagnostic tests have undergone evaluation in more research studies.

Another reason for the performance differences between the two markers could be that Rili-BÄK stipulates mandatory participation in CRP-EQAs in Germany while it is mainly voluntary for PCT [13]. Participation in EQAs is a useful tool for identifying potential problems in the laboratory process and provides an independent overview of the current testing devices. Our observation of the performance of individual laboratories supports this hypothesis, as the overall performance of the four CRP laboratories was more stable over the observed period of time than the performance of the three PCT laboratories. In addition, our results once again confirm the benefits of external quality testing of laboratory performance, as individual participants achieved better and more comparable results in the EQAs following a false EQA result. Therefore, we recommend that EQAs become mandatory for PCT to further improve the diagnostic strength of this marker.

Another way to improve the diagnostic accuracy of the test systems would certainly be to develop reference measurement procedures that would provide a reliable system for generating calibrators with a high accuracy and a low uncertainty of defined magnitude [32, 33]. Since 2001, an IFCC-certified reference material and a protocol for reference methods based on immunoturbidimetry or nephelometry [34] have been established for CRP. Nevertheless, they do not seem to have been fully evaluated or accepted, since RiliBÄK still recommends the use of a method-specific target value instead of the reference method value for the evaluation of CRP-EQAs [13]. Furthermore, a recent publication by Wu et al. tested the ICFF CRP reference material ERM-DA474 in five different analytical assays and found significant variations between these tests even after recalibration [35]. This analysis is an additional indication that higher accuracy is needed to distinguish between minor changes and to thus increase the diagnostic strength of the test systems. Furthermore, current CRP test methods can no longer be considered to be cutting-edge metrological procedures since protein analysis and quantification via mass spectrometry are already possible.

There are a few publications that compare the current detection methods to mass-spectrometric analysis. Williams et al. showed that the immunometric assays and a one-peptide based mass spectrometric approach presented at least good correlation despite a value difference of one order of magnitude [36]. Alternatively, isotope diluted mass spectrometry (IDMS) is frequently used as a reference measurement procedure, particularly for small molecules and analytes [37]. We have already been able to show the applicability of an LC-IDMS approach for HbA1c [38] and Kilpatrick and Bunk revealed promising results for an LC-MS/MS approach for detecting CRP using an affinity purifying procedure [33]. They have also successfully generated a ^(15)^N-CRP as an internal standard for IDMS [39], but unfortunately research on reference measurement methods have yet to yield any new information.

Unfortunately, there is currently no reference method for detecting PCT in serum. This is certainly another reason why results are more comparable for CRP in our EQAs.

Taken together, the EQA results for CRP show an overall comparability of the median results even though the immunological methods are still in need of some improvement. For PCT, we observed a manufacturer-dependent difference in median values of around 27% for manufacturer r014 compared to the rest of the participants. The distribution of values can still be improved for both parameters, as indicated by the coefficient of variation. Unfortunately, it is not possible to indicate a method with the highest accuracy for either analyte, since a reference measurement method for PCT is still outstanding and the currently accepted method for CRP is no longer a cutting-edge metrological process. We recommend stepping up research on reference measurement methods for both analytes to enhance the accuracy and diagnostic strength of the test systems. Furthermore, participation in PCT-EQAs should become mandatory as well in order to further improve the general quality of individual laboratories. This might only have a small impact on an individual patient but is most certainly of utmost importance for comparing and validating clinical studies, especially on a multi-center basis.

## Supporting information

S1 Table Method-specific sorted CRP EQA results. This table contains the raw results of the EQA participants without any correction. Results from obvious swap of samples are highlighted in green. Results that were excluded because they exceede the calibration by > 20% in at least one sample are highlighted in orange.
(XLSX)

S2 Table Manufacturer-specific sorted PCT EQA results. This table contains the raw results of the EQA participants without any correction. Results from obvious swap of samples are highlighted in green. Results that were excluded because they exceede the calibration by > 20% in at least one sample are highlighted in orange.
(XLSX)

S3 Table Comparison of the value distribution of CRP and PCT measurements of single laboratories in comparison to the median result of all participants. The legend for the different colours are indicated in the corresponding table sheets.
(XLSX)

## References

[1] Plebani M, Laposata M, Lundberg GD. The brain-to-brain loop concept for laboratory testing 40 years after its introduction. American journal of clinical pathology. 2011;136(6):829–33. 10.1309/AJCPR28HWHSSDNON 22095366

[2] Fan SL, Miller NS, Lee J, Remick DG. Diagnosing sepsis—The role of laboratory medicine. Clinica chimica acta; international journal of clinical chemistry. 2016;460:203–10. 10.1016/j.cca.2016.07.002 27387712 PMC4980259

[3] Zhydkov A, Christ-Crain M, Thomann R, Hoess C, Henzen C, Werner Z, et al Utility of procalcitonin, C-reactive protein and white blood cells alone and in combination for the prediction of clinical outcomes in community-acquired pneumonia. Clinical chemistry and laboratory medicine. 2015;53(4):559–66. 10.1515/cclm-2014-0456 25014522

[4] Lobo SM. Sequential C-reactive protein measurements in patients with serious infections: does it help? Critical care (London, England). 2012;16(3):130. 10.1186/CC11347 PMC3580631 22731851

[5] Povoa P. C-reactive protein: a valuable marker of sepsis. Intensive care medicine. 2002;28(3):235–43. 10.1007/s00134-002-1209-6 11904651

[6] Povoa P, Almeida E, Moreira P, Fernandes A, Mealha R, Aragao A, et al C-reactive protein as an indicator of sepsis. Intensive care medicine. 1998;24(10):1052–6. 9840239 10.1007/s001340050715

[7] Henriquez-Camacho C, Losa J. Biomarkers for sepsis. BioMed research international. 2014;2014:547818 10.1155/2014/547818 24800240 PMC3985161

[8] Jaye DL, Waites KB. Clinical applications of C-reactive protein in pediatrics. The Pediatric infectious disease journal. 1997;16(8):735–46; quiz 46–7. 9271034 10.1097/00006454-199708000-00003

[9] Meisner M. Update on procalcitonin measurements. Annals of laboratory medicine. 2014;34(4):263–73. 10.3343/alm.2014.34.4.263 24982830 PMC4071182

[10] Povoa P, Teixeira-Pinto AM, Carneiro AH. C-reactive protein, an early marker of community-acquired sepsis resolution: a multi-center prospective observational study. Critical care (London, England). 2011;15(4):R169. 10.1186/cc10313 PMC3387609 21762483

[11] Seligman R, Meisner M, Lisboa TC, Hertz FT, Filippin TB, Fachel JM, et al Decreases in procalcitonin and C-reactive protein are strong predictors of survival in ventilator-associated pneumonia. Critical care (London, England). 2006;10(5):R125. 10.1186/cc5036 PMC1751074 16956405

[12] Epner PL, Gans JE, Graber ML. When diagnostic testing leads to harm: a new outcomes-based approach for laboratory medicine. BMJ quality & safety. 2013;22 Suppl 2:ii6–ii10. 10.1136/bmjqs-2012-001621 PMC3786651 23955467

[13] Richtlinie der Bundesärztekammer zur Qualitätssicherung laboratoriumsmedizinischer Untersuchungen: RiLiBÄK. Deutsches Ärzteblatt. 2014;111(38):1583–618.

[14] Müller I, Besier S, Hintereder G, Brade V, Hunfeld K. Zur Qualität der bakteriologischen Infektionsserologie in Deutschland: eine Metaanalyse der infektionsserologischen Ringversuche des Jahres 2006—Beitrag der Qualitätssicherungskommission der DGHM. GMS Z Forder Qualitatssich Med Lab. 2009;1:1–21.

[15] DIN E. ISO/IEC 17043: 2010–05: Konformitätsbewertung-Allgemeine Anforderungen an Eignungsprüfungen (ISO/IEC 17043: 2010). Deutsche und Englische Fassung EN ISO/IEC. 2010;17043.

[16] Guder WG, Nolte J. Das Laborbuch: für Klinik und Praxis: Elsevier GmbH, Der Urban & Fischer Verlag; 2005.

[17] Thomas L. Labor und Diagnose: Indikation und Bewertung von Laborbefunden für die medizinische Diagnostik. Frankfurt/Main: Th-Books-Verl.-Ges.; 2012.

[18] O'Grady NP, Barie PS, Bartlett JG, Bleck T, Carroll K, Kalil AC, et al Guidelines for evaluation of new fever in critically ill adult patients: 2008 update from the American College of Critical Care Medicine and the Infectious Diseases Society of America. Critical care medicine. 2008;36(4):1330–49. 10.1097/CCM.0b013e318169eda9 18379262

[19] Sager R, Kutz A, Mueller B, Schuetz P. Procalcitonin-guided diagnosis and antibiotic stewardship revisited. BMC medicine. 2017;15(1):15 10.1186/s12916-017-0795-7 28114931 PMC5259962

[20] Ljungstrom L, Pernestig AK, Jacobsson G, Andersson R, Usener B, Tilevik D. Diagnostic accuracy of procalcitonin, neutrophil-lymphocyte count ratio, C-reactive protein, and lactate in patients with suspected bacterial sepsis. PloS one. 2017;12(7):e0181704 10.1371/journal.pone.0181704 28727802 PMC5519182

[21] Nora D, Salluh J, Martin-Loeches I, Povoa P. Biomarker-guided antibiotic therapy-strengths and limitations. Annals of translational medicine. 2017;5(10):208 10.21037/atm.2017.04.04 28603723 PMC5451622

[22] Tang BM, Eslick GD, Craig JC, McLean AS. Accuracy of procalcitonin for sepsis diagnosis in critically ill patients: systematic review and meta-analysis. The Lancet Infectious diseases. 2007;7(3):210–7. 10.1016/S1473-3099(07)70052-X 17317602

[23] Branche A, Neeser O, Mueller B, Schuetz P. Procalcitonin to guide antibiotic decision making. Curr Opin Infect Dis. 2019. 10.1097/QCO.0000000000000522 30648993

[24] Algarra M, Gomes D, Esteves da Silva JC. Current analytical strategies for C-reactive protein quantification in blood. Clinica chimica acta; international journal of clinical chemistry. 2013;415:1–9. 10.1016/j.cca.2012.09.007 22975530

[25] Wojtalewicz N, Goseberg S, Kabrodt K, Schellenberg I. Six years of INSTAND e. V. sIgE proficiency testing: An evaluation of in vitro allergy diagnostics. Allergo J Int. 2017;26(2):43–52. 10.1007/s40629-016-0005-8 28344920 PMC5346112

[26] Wojtalewicz N, Kabrodt K, Goseberg S, Schellenberg I. Evaluation of the manufacturer-dependent differences in sIgE results for indoor allergens. Annals of allergy, asthma & immunology: official publication of the American College of Allergy, Asthma, & Immunology. 2018. 10.1016/j.anai.2018.07.016 30025909

[27] de Wolf HK, Gunnewiek JK, Berk Y, van den Ouweland J, de Metz M. Comparison of a new procalcitonin assay from roche with the established method on the brahms kryptor. Clinical chemistry. 2009;55(5):1043–4. 10.1373/clinchem.2008.117655 19264851

[28] Prieto B, Alvarez FV. Lack of transferability of results between procalcitonin assays. Clinical chemistry. 2009;55(12):2226–7; author reply 7–8. 10.1373/clinchem.2009.132605 19797714

[29] Soh A, Binder L, Clough M, Hernandez MH, Lefevre G, Mostert K, et al Comparison of the novel ARCHITECT procalcitonin assay with established procalcitonin assay systems. Practical laboratory medicine. 2018;12:e00110 10.1016/j.plabm.2018.e00110 30519621 PMC6249413

[30] Meisner M, Lohs T, Huettemann E, Schmidt J, Hueller M, Reinhart K. The plasma elimination rate and urinary secretion of procalcitonin in patients with normal and impaired renal function. European journal of anaesthesiology. 2001;18(2):79–87. 11270029 10.1046/j.0265-0215.2000.00783.x

[31] Schuetz P, Bretscher C, Bernasconi L, Mueller B. Overview of procalcitonin assays and procalcitonin-guided protocols for the management of patients with infections and sepsis. Expert review of molecular diagnostics. 2017;17(6):593–601. 10.1080/14737159.2017.1324299 28443360

[32] Bunk DM. Reference materials and reference measurement procedures: an overview from a national metrology institute. The Clinical biochemist Reviews. 2007;28(4):131–7. 18392127 PMC2282405

[33] Kilpatrick EL, Bunk DM. Reference measurement procedure development for C-reactive protein in human serum. Analytical chemistry. 2009;81(20):8610–6. 10.1021/ac901597h 19764742

[34] Blirup-Jensen S. Protein standardization III: Method optimization basic principles for quantitative determination of human serum proteins on automated instruments based on turbidimetry or nephelometry. Clinical chemistry and laboratory medicine. 2001;39(11):1098–109. 10.1515/CCLM.2001.175 11831625

[35] Wu C, Zhang S, Liu W, Zeng J, Zhao T, Yue Y, et al Application of Commutable ERM-DA474/IFCC for Harmonization of C-reactive Protein Measurement Using Five Analytical Assays. Clinical laboratory. 2017;63(11):1883–8. 10.7754/Clin.Lab.2017.170626 29226658

[36] Williams DK, Muddiman DC. Absolute quantification of C-reactive protein in human plasma derived from patients with epithelial ovarian cancer utilizing protein cleavage isotope dilution mass spectrometry. Journal of proteome research. 2009;8(2):1085–90. 10.1021/pr800922p 19196186 PMC2637469

[37] Copeland BE, Grisley DW, Casella J, Bailey H. Comparison of serum calcium measurements with respect to five models of atomic absorption spectrometers using NBS-AACC calcium reference method and isotope-dilution mass spectrometry as the definitive method. American journal of clinical pathology. 1976;66(4):619–33. 10.1093/ajcp/66.4.619 788497

[38] Kaiser P, Akerboom T, Ohlendorf R, Reinauer H. Liquid chromatography-isotope dilution-mass spectrometry as a new basis for the reference measurement procedure for hemoglobin A1c determination. Clinical chemistry. 2010;56(5):750–4. 10.1373/clinchem.2009.139477 20299680

[39] Kilpatrick EL, Liao WL, Camara JE, Turko IV, Bunk DM. Expression and characterization of 15N-labeled human C-reactive protein in Escherichia coli and Pichia pastoris for use in isotope-dilution mass spectrometry. Protein expression and purification. 2012;85(1):94–9. 10.1016/j.pep.2012.06.019 22796447

